# *Pseudomonas aeruginosa* exotoxin A is not essential for corneal disease severity or bacterial survival

**DOI:** 10.1128/iai.00165-26

**Published:** 2026-07-22

**Authors:** James D. Begando, Michaela E. Marshall, Emma Caitlin Kundracik, George R. Dubyak, Arne Rietsch, Eric Pearlman

**Affiliations:** 1 Department of Physiology and Biophysics, University of California 197206, Irvine, California, USA; 2 Department of Molecular Biology and Microbiology, Case Western Reserve University 2546, Cleveland, Ohio, USA; 3 Department of Physiology and Biophysics, Case Western Reserve University 2546, Cleveland, Ohio, USA; 4 Department of Ophthalmology, University of California 481087, Irvine, California, USA; University of California San Diego School of Medicine, La Jolla, California, USA

**Keywords:** inflammation, neutrophils, keratitis, cornea, Exotoxin A (ToxA), *Pseudomonas aeruginosa*

## Abstract

*Pseudomonas aeruginosa* produces multiple toxins and exoenzymes that contribute to its survival and ability to cause disease. In the current study, we examined whether the type II-secreted cytotoxin Exotoxin A (ToxA) is required for *P. aeruginosa* growth and disease severity in infected murine corneas. Using Δ*toxA* mutants and complemented strains on a PAO1 background, we report that although ToxA is produced during corneal infection, ToxA deletion did not significantly affect bacterial replication, neutrophil recruitment, or disease severity in infected corneas. These findings contrast with an earlier study identifying a role for ToxA in *P. aeruginosa* keratitis.

## INTRODUCTION

*Pseudomonas aeruginosa* is a major cause of corneal blindness in the United States and worldwide, with major risk factors being ocular injury and poor contact lens hygiene (1, 2). Corneal ulcers from patients infected with pathogenic fungi or bacteria, including *P. aeruginosa*, have a predominant neutrophil infiltrate, and elevated pro-inflammatory and chemotactic cytokines (3, 4). We and others have used murine models of *P. aeruginosa* keratitis to show that infection induces epithelial injury and inflammatory cytokine production, promoting neutrophil recruitment into the corneal stroma. Although neutrophils are required for bacterial control, these cells also release granule proteases that cause corneal tissue damage resulting in increased disease severity (5–7).

In addition to host-mediated inflammation*, P. aeruginosa* produces multiple virulence factors that contribute to corneal pathogenesis. We and others demonstrated that *P. aeruginosa* type III secretion system (T3SS) plays a major role in the pathogenesis of *P. aeruginosa* keratitis (8–11). In addition to the T3SS, *P. aeruginosa* produces several other virulence factors that can damage host tissues and contribute to disease. Among these is Exotoxin A (ToxA), which is secreted through the type II secretion system (T2SS). Unlike the T3SS, which injects effector proteins directly into host cells, the T2SS exports toxins into the extracellular environment, where they can target host cells.

In the current study, we use Δ*toxA* mutants and complemented strains to show that ToxA is not required for bacterial survival or corneal disease severity during *P. aeruginosa* keratitis.

## MATERIALS AND METHODS

### Construction of *toxA* reporter strain

The *toxA* promoter reporter plasmid was constructed by replacing GFPmut3 in pP33-*p_exoS_*-GFP with a synthesized, codon-optimized superfolder GFP (Green Fluorescence Protein, GFPsf; BioBasic). The *p_exoS_* promoter was subsequently replaced with the *toxA* promoter amplified from PAO1F genomic DNA. The parental pP33-*p_exoS_*-GFP plasmid was described previously (12). The *toxA* reporter strain carried a *toxA* promoter-GFP reporter plasmid, which also confers gentamicin resistance. A second plasmid was included that encodes a constitutive mCherry red fluorescence protein in addition to a gene that confers carbenicillin resistance. Gentamicin and carbenicillin were included in overnight growth medium.

### Construction of Δ*toxA* and complemented strains

An in-frame deletion of *toxA* was generated in the parent PAO1F strain by allelic exchange using the vector pEXG2 as previously described (13). Regions flanking *toxA* were amplified from genomic DNA and assembled into pEXG2, and the resulting construct was introduced into PAO1F by conjugation. Double-crossover mutants were isolated by gentamicin selection followed by sucrose counterselection, and deletion of *toxA* was confirmed by PCR.

For complementation, the *toxA* open reading frame with its native promoter was cloned into the mini-Tn7 delivery vector pUC18-miniTn7T-Gm and integrated at the chromosomal Tn7 attachment site using the Tn7 transposition system as previously described (14). The antibiotic resistance cassette from the Δ*toxA::toxA* strain was subsequently excised using FLP recombinase.

Additionally, the PAO1F, T3SS-low expressing strain PAO1 RP289 (15, 16), and Δ*pscD* strains used for infections carried the constitutive GFP plasmid pP25-GFPo, which confers carbenicillin resistance. Carbenicillin was included when strains were streaked and grown overnight to maintain plasmids but was omitted after back-dilution during the preparation of bacteria for infection experiments.

### Low T3SS and T3SS mutant strains

We also generated ∆*toxA* mutant in PAO1 strains that express low levels of T3SS and in the ∆*pscD* T3SS deletion mutant. The low T3SS strain is RP289, which exibits T3SS-dependent cell cytotoxicity (15, 16). We show here that this strain had no detectable ExoT secretion by immunoblot under the conditions tested (Fig. S4A). This allowed us to test whether ToxA contributes to disease in a background where T3SS activity is low, but can still cause infection compared with ∆*pscD*. Consistent with this phenotype, ExoT was detected in PAO1F supernatants under low-Ca^2+^ conditions but was not detectable in Δ*pscD* or T3SS-low supernatants (Fig. S4A). We further measured exoS promoter activity using reporter strains in which lacZ expression is driven by the exoS promoter. β-Galactosidase activity was increased in PAO1F under low-Ca^2+^ conditions but remained lower in Δ*pscD* and the T3SS-low strains (Fig. S4B). ToxA deletion was also confirmed in the corresponding Δ*toxA* strains (Fig. S4C).

### Growth conditions

*P. aeruginosa* strains were cultured at 37°C with shaking in dialyzed, Chelex-tryptic soy broth dialysate-free (TSB-DF) supplemented with 1% glycerol and 100 mM monosodium glutamate, adapted from previously described conditions for induction of ToxA expression and keratitis infection models (17, 18). Overnight cultures were sub-cultured to late-log phase (OD_600_ ≈ 0.9), washed, and resuspended in PBS prior to infection.

### Mouse corneal infection model

C57BL/6 mice were purchased from the Jackson Laboratory and bred in-house. Male and female mice ages 6–12 weeks were used for experiments. All procedures were approved by the University of California, Irvine Institutional Animal Care and Use Committee.

Overnight cultures of *P. aeruginosa* PAO1F or mutant strains were grown in TSB-DF, sub-cultured to late-log phase (OD_600_ ≈ 0.9), washed, and resuspended in PBS. C57BL/6 mice were anesthetized with ketamine/xylazine, the corneal epithelium was abraded with three parallel scratches using a sterile 26-gauge needle, and 2 µL of bacterial suspension (~1 × 10^6^ CFU/eye) was applied topically as previously described (9, 19).

### *In vivo* imaging and bacterial growth (CFU assay)

At 24h or 48h post-infection, corneas were imaged by brightfield microscopy to assess opacity or by fluorescence microscopy to detect GFP or mCherry-expressing bacteria. For bacterial growth, whole eyes were homogenized in PBS using a TissueLyser II (Qiagen), serially diluted, and plated on LB agar. Colony-forming units (CFU) were counted manually. Images of infected mouse eyes were acquired using a Leica MZ10 F stereomicroscope equipped with a Leica DFC450 C digital camera.

### Corneal opacity and fluorescence quantification

Corneal opacity and fluorescence were quantified from digital eye images using custom ImageJ macros. For each analysis, images were converted to 8-bit grayscale, and a circular region of interest was automatically centered over the central cornea. Pixels outside the corneal region of interest were excluded from analysis.

For corneal opacity quantification, saturated glare pixels were excluded from both the numerator and denominator. Valid corneal pixels were defined as the total number of pixels within the corneal region of interest after exclusion of glare pixels. Opaque pixels were defined as pixels within the selected opacity threshold range, excluding the glare range. Percent corneal opacity was calculated as: Percent corneal opacity = (opaque pixels/valid corneal pixels) × 100.

For GFP and mCherry fluorescence analysis, fluorescence-positive pixels were defined using a fixed grayscale threshold range, excluding saturated glare pixels. Because infected corneas contained both focal high-intensity fluorescence and broader low-intensity signal, fluorescence was quantified as the sum of squared grayscale intensity values across all fluorescence-positive pixels within the corneal region of interest: Σ(pixel intensity)². This approach separates visually distinct fluorescence patterns that can produce similar linear integrated density values. The same threshold, region of interest, glare exclusion, and quantification parameters were applied across all images within each analysis.

### Immunoblot analysis of bacterial lysates and culture supernatants

PAO1F, Δ*toxA*, and complemented strains were grown overnight in TSB-DF, sub-cultured the following day, and grown to late-log phase (OD_600_ ≈ 0.9). Cultures were separated into cell pellet and supernatant fractions by centrifugation at 3,000 × *g* for 10 min. Supernatants were filtered through 0.22 µm syringe filters to remove bacteria, and equal culture volumes were concentrated by trichloroacetic acid (TCA) precipitation. TCA was added to filtered supernatants to a final concentration of 10%; samples were incubated on ice, and precipitated proteins were collected by centrifugation at 14,000 × *g* for 10 min at 4°C. Protein pellets were washed with cold acetone, air-dried, and resuspended in 1× SDS sample buffer.

Proteins were then resolved on 10% SDS-PAGE gels, transferred to PVDF membranes, and probed with anti-ToxA (Sigma-Aldrich, P2318; 1:5,000), anti-RpoA (BioLegend Cat #663104, 1:10,000), or an affinity-purified rabbit polyclonal anti-ExoT antibody previously described by Lee et al. (20). Fluorescent secondary antibodies were used for detection (anti-rabbit IRDye-800 for ToxA and anti-mouse IRDye-680 for RpoA).

### Immunoprecipitation and immunoblot analysis of corneal lysates

C57BL/6 mice were infected as described above, and corneas were dissected 24h post-infection. Four infected corneas were pooled from each group and homogenized in denaturing lysis buffer (50 mM Tris-HCl pH 7.5, 150 mM NaCl, 1% SDS, 1 mM EDTA, and protease inhibitors), heated, and clarified by centrifugation at 14,000 × *g* for 10 min. Lysates were diluted 10-fold in lysis buffer lacking SDS to reduce the SDS concentration before immunoprecipitation and incubated overnight at 4°C with anti-ToxA antibody pre-bound to Protein G magnetic beads. Beads were washed with Tris-buffered saline, followed by a high-salt wash containing 500 mM NaCl to reduce nonspecific binding, and bound proteins were eluted by heating in reducing Laemmli sample buffer.

Eluates were resolved by SDS-PAGE, transferred to PVDF membranes, and probed overnight at 4°C with anti-ToxA antibody (Sigma-Aldrich, P2318), followed by HRP-conjugated anti-rabbit secondary antibody (Cell Signaling Technology, #7074). Signal was detected by chemiluminescence.

### Human corneal epithelial cell cytotoxicity assay

The human telomerase-immortalized corneal epithelial cell line (hTCEpi) was kindly provided by Dr. Suzanne Fleiszig, University of California, Berkeley, and maintained in keratinocyte growth medium (KGM; Lonza) supplemented with KGM SingleQuots. Cells were grown at 37°C until approximately 80% confluent, washed with warm PBS, and detached using 0.05% trypsin-EDTA. Trypsin was neutralized with KGM, and cells were collected by centrifugation at 150 × *g* for 5 min. Cells were resuspended in KGM, counted using a hemocytometer, and seeded into 96-well plates at 3 × 10^4^ cells per well.

*P. aeruginosa* culture supernatants were prepared from overnight cultures grown in TSB-DF. Bacteria were removed by centrifugation at 5,000 × *g* for 5 min, followed by filtration of the supernatants through 0.22 µm syringe filters. Filtered bacterial cultures from PAO1F, Δ*toxA*, or Δ*toxA::toxA* strains were added to hTCEpi cultures at a final concentration of 10% in KGM. hTCEpi cells were incubated overnight, and cell lysis was quantified using the CytoTox 96 Non-Radioactive Cytotoxicity Assay (Promega) according to the manufacturer’s instructions. This assay measures LDH activity in culture supernatants following cell lysis.

### Flow cytometry

To recover cells, infected corneas were dissected and incubated at 37°C for 45–60 min in collagenase digestion buffer, using 500 µL per cornea. The digestion buffer comprised RPMI supplemented with 10% FBS, 1× penicillin-streptomycin, 1 × MEM non-essential amino acids (Gibco, 11140-050), 20–25 mM HEPES buffer solution (Gibco, 15630-080), 2 mM sodium pyruvate (Gibco, 11360-070), DNase I (~0.125 mg/mL), and collagenase type I from *Clostridium histolyticum* (5 mg/mL; Sigma-Aldrich, C0130). Samples were gently agitated every 15 min to promote tissue digestion.

Collagenase digestion was stopped by quenching with FBS, and cells were filtered through a 70 μm strainer to remove non-cellular debris, pelleted by centrifugation (500 × *g*), and resuspended in flow cytometry buffer.

All subsequent steps were performed at 4°C. Cells were incubated with anti-CD16/32 (BioLegend) for 10 min to block Fc receptors, followed by staining with the fixable viability dye eFluor780 and fluorophore-conjugated antibodies against CD45 (Bio-Rad Laboratories, Inc., StarBright Violet 670), CD11b (Bio-Rad Laboratories, Inc., StarBright Violet 790), Ly6C (Bio-Rad Laboratories, Inc., StarBright 575), CCR2 (BioLegend, APC), and Ly6G (BioLegend, Brilliant Violet 421). Cells were fixed using BD Cytofix/Cytoperm fixation/permeabilization solution (BD Biosciences, 554722), washed, and analyzed on an ACEA NovoCyte flow cytometer using NovoExpress software. Cells were gated as singlets and live cells, followed by CD45^+^CD11b^+^ myeloid cells. Neutrophils were identified as Ly6G^+^ and inflammatory monocytes as Ly6C^+^CCR2^+^ cells.

### Statistical analysis

Statistical analyses were performed using GraphPad Prism. For comparisons involving more than two groups, statistical significance was determined by one-way ANOVA followed by Dunnett’s multiple comparisons test. Differences were considered significant when *P* < 0.05.

## RESULTS

### ToxA is expressed in the cornea during PAO1F infection

**Fig 1:**
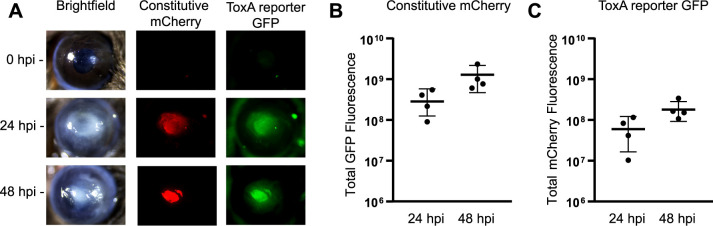
*In vivo* expression of ToxA during *P. aeruginosa* corneal infection. (**A**) Representative brightfield, constitutive mCherry, and *toxA* promoter-driven GFP fluorescence images of corneas infected with a *toxA* reporter strain at 0h, 24h, and 48h post-infection. mCherry indicates total bacteria, and GFP represents *toxA* promoter activity. (**B**) Constitutive bacterial mCherry fluorescence quantified from infected corneas. (**C**) GFP fluorescence quantified as a measure of *toxA* expression. Each data point represents a single cornea. Error bars represent mean ± SD.

To quantify ToxA expression *in vivo*, mice were infected with a PAO1F strain that expresses GFP under the *toxA* promoter and constitutively expresses mCherry. Corneas were examined 24h and 48h post-infection. Constitutive mCherry fluorescence indicates bacterial localization within the cornea, while GFP fluorescence shows ToxA promoter activity. Both mCherry and GFP were detected at 24h post-infection and increased after 48h (Fig. 1A through C). Control imaging of single-fluorophore strains confirmed that GFP and mCherry signals were detected in separate channels without fluorescence bleed-through (Fig. S1). These findings demonstrate that ToxA is expressed in corneas infected with *P. aeruginosa*.

### No difference in *P. aeruginosa* growth or corneal disease severity in the absence of ToxA

**Fig 2:**
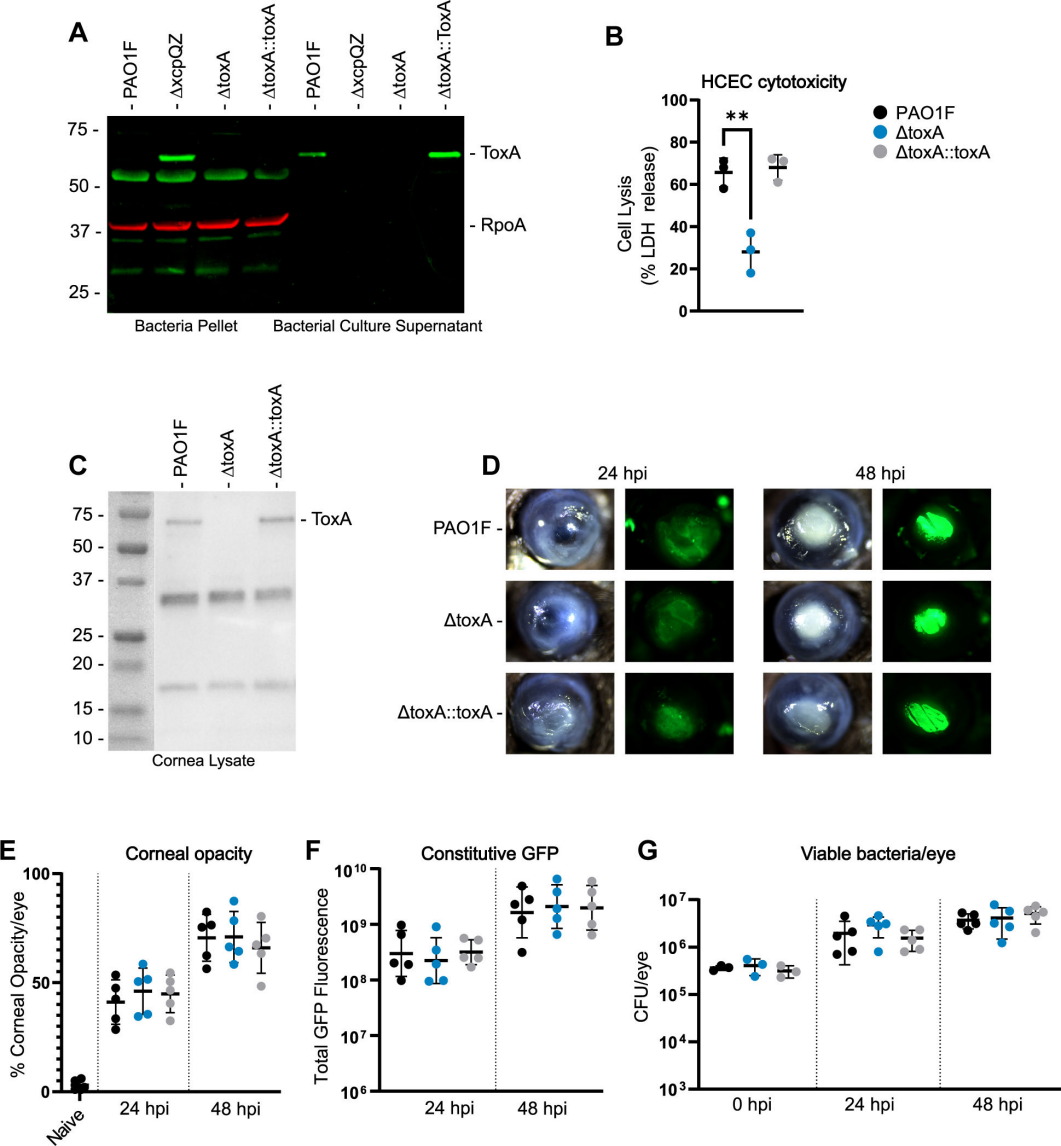
Corneal infection with PAO1F ToxA mutants. (**A**) Immunoblot detection of ToxA in bacterial pellet and bacterial culture medium from the indicated strains. ToxA is present in PAO1F and the complemented strain, but absent in Δ*toxA*. The Δ*xcpQZ* strain retains ToxA in the bacterial pellet, consistent with impaired type II secretion. RpoA serves as a loading control. (**B**) Cell lysis of hTCEpi cells treated with filtered bacterial culture supernatants from PAO1F, Δ*toxA*, or Δ*toxA::toxA* strains, measured by LDH release. (**C**) ToxA protein in corneal lysates by immunoblotting 24h post-infection with the indicated strains. (**D**) Representative brightfield and constitutive bacterial GFP fluorescence images of infected corneas at 24h and 48h post-infection. (**E**) Corneal opacity quantified and expressed as a percentage. (**F**) Constitutive bacterial GFP fluorescence quantified from infected corneas. (**G**) Viable bacteria from homogenized eyes quantified by colony-forming unit (CFU). Each data point in panel B represents an independent biological replicate. Data points in panels **E–G** are individual infected eyes. Error bars represent mean ± SD. Statistical analysis was performed using one-way ANOVA. **P<0.01.

To determine if there is a role for ToxA in corneal disease severity, we generated a Δ*toxA* mutant and a complemented strain on a PAO1F background. Immunoblot analysis of TCA precipitated growth medium following overnight growth confirmed the presence of ToxA protein in the parent PAO1F and in the complemented strain, and its absence in the Δ*toxA* mutant (Fig. 2A). As a secretion control, a Δ*xcpQZ* mutant was used which retained detectable ToxA in the bacterial pellet but not in medium, consistent with the expected impaired secretion of ToxA (Fig. 2A). Production of the ribosomal protein RpoA confirmed similar total protein levels in each well.

As a functional validation of the Δ*toxA* and complemented Δ*toxA::toxA* strains, human corneal epithelial cells were incubated 18 h with bacterial culture supernatants from parental PAO1F, Δ*toxA*, or Δ*toxA::toxA*. Conditioned medium from PAO1F and Δ*toxA::toxA* induced >60% hCEC death, whereas conditioned medium from the Δ*toxA* mutant caused significantly less cell death (Fig. 2B), confirming loss and restoration of ToxA-dependent cytotoxic activity.

To assess the role of ToxA in *P. aeruginosa* keratitis, abraded corneas of C57BL/6 mice were infected topically with PAO1F, the Δ*toxA* mutant, or Δ*toxA::toxA*, and evaluated 24h and 48h after infection. To confirm ToxA production during infection, corneas were dissected 24h post-infection, and lysates were subjected to ToxA immunoprecipitation and immunoblotting. We found ToxA in corneas infected with PAO1F and Δ*toxA::toxA*, but not those infected with Δ*toxA* (Fig. 2C).

PAO1F caused a severe, rapidly progressive corneal infection, with constitutive bacterial GFP fluorescence detected in infected corneas (Fig. 2D). However, we found no significant differences in corneal opacification in mice infected with PAO1F, Δ*toxA*, or Δ*toxA::toxA* (Fig. 2D and E). Similarly, there was no significant difference in total GFP fluorescence (Fig. 2D and F). Viable bacteria in corneas infected with PAO1F, Δ*toxA*, or Δ*toxA::toxA* quantified by CFU were also not significantly different at 24h or 48h post-infection (Fig. 2G). All brightfield and GFP images in this experiment are shown in Fig. S2. Taken together, these findings indicate that ToxA does not regulate bacterial growth or corneal disease severity.

### ToxA does not regulate host inflammatory cell recruitment

**Fig 3:**
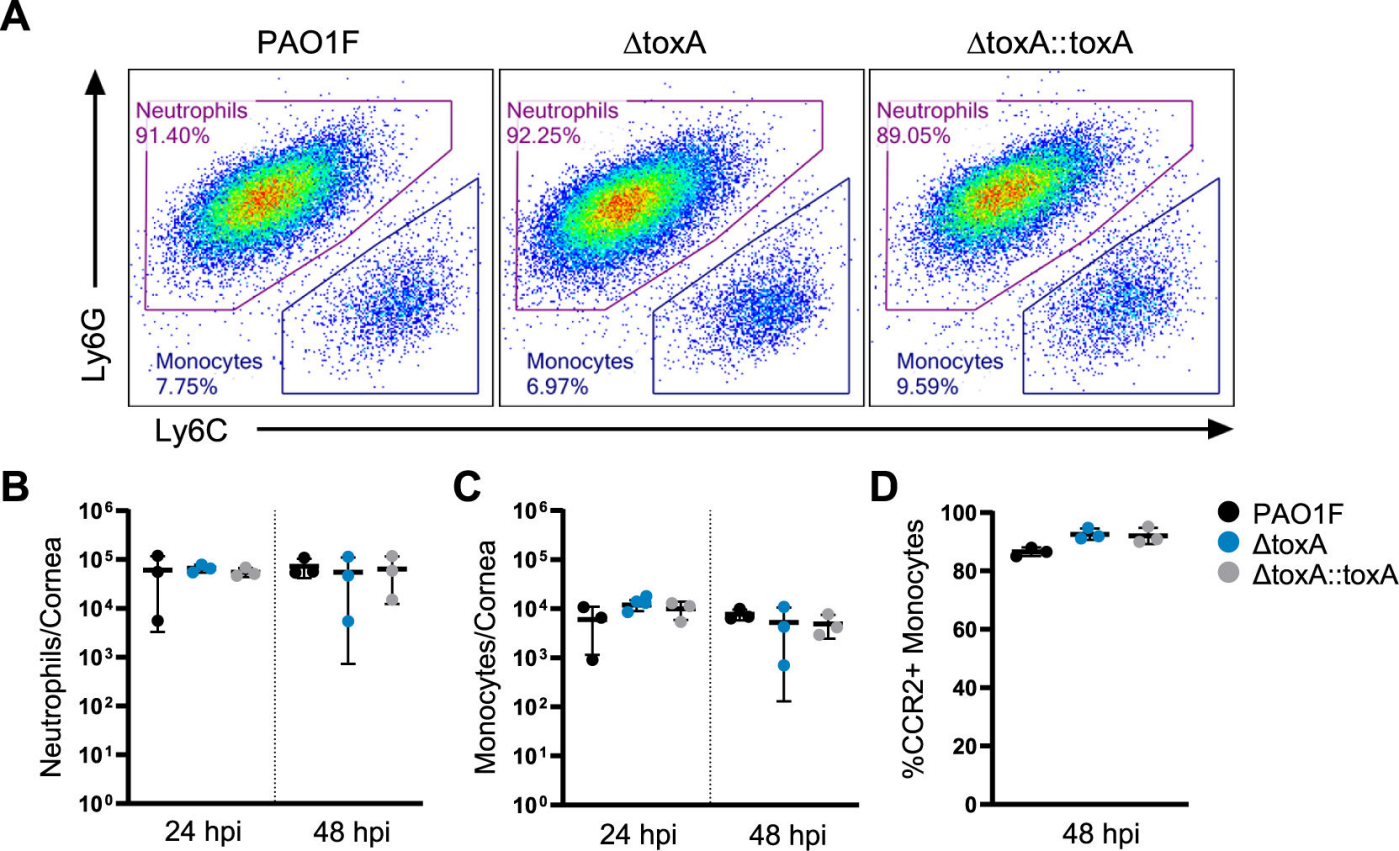
Neutrophil and monocyte recruitment to infected corneas. (**A**) Representative flow cytometry scatter plots from collagenase-digested corneas 24h post-infection with PAO1F, Δ*toxA*, or Δ*toxA::toxA P. aeruginosa*. Cells were incubated with Ly6G and Ly6C to identify neutrophils (Ly6G^+^) and monocytes (Ly6C^+^). Percentages represent frequencies within the CD45^+^/CD11b^+^ population. (**B**) Total number of neutrophils per cornea at 24h and 48h post-infection. (**C**) Total number of monocytes per cornea at 24h and 48h post-infection. (**D**) Percentage of monocytes expressing CCR2 (inflammatory monocytes) at 48h post-infection. Each data point represents a single cornea. Error bars represent mean ± SD. Statistical analysis was performed using one-way ANOVA. *P* > 0.05 for all comparisons.

To quantify leukocyte infiltration, we performed flow cytometry at 24h post-infection and found that approximately 90% of infiltrating CD45^+^CD11b^+^ cells were neutrophils and ~10% were monocytes (Fig. 3A), consistent with our findings in infected patients and mice (4, 19). The gating strategy used to identify live CD45^+^CD11b^+^ myeloid cells, Ly6G^+^ neutrophils, and Ly6C^+^ monocytes is shown in Fig. S3. The number of neutrophils and monocytes was not significantly different among these groups (Fig. 3B and C). After 48h, ~90% of monocytes expressed CCR2, consistent with an inflammatory monocyte phenotype (19), and the percent inflammatory monocytes was not significantly different among PAO1F, Δ*toxA*, and Δ*toxA::toxA* (Fig. 3D). Overall, inflammatory cell recruitment was not affected by the absence of ToxA.

### ToxA is not required for corneal disease in strains with reduced or no T3SS production

To determine whether T3SS activity masked a contribution of ToxA, we repeated these experiments in strains with reduced T3SS activity or absent T3SS exoenzyme secretion. Because the PAO1F background exhibits high T3SS activity that can dominate corneal pathology, we tested whether lowering or eliminating T3SS would reveal a ToxA-dependent phenotype. We used PAO1 strain RP289, which exhibits reduced and delayed type III secretion gene expression, producing weaker and slower T3SS-dependent phenotypes *in vitro* (15, 16).

**Fig 4:**
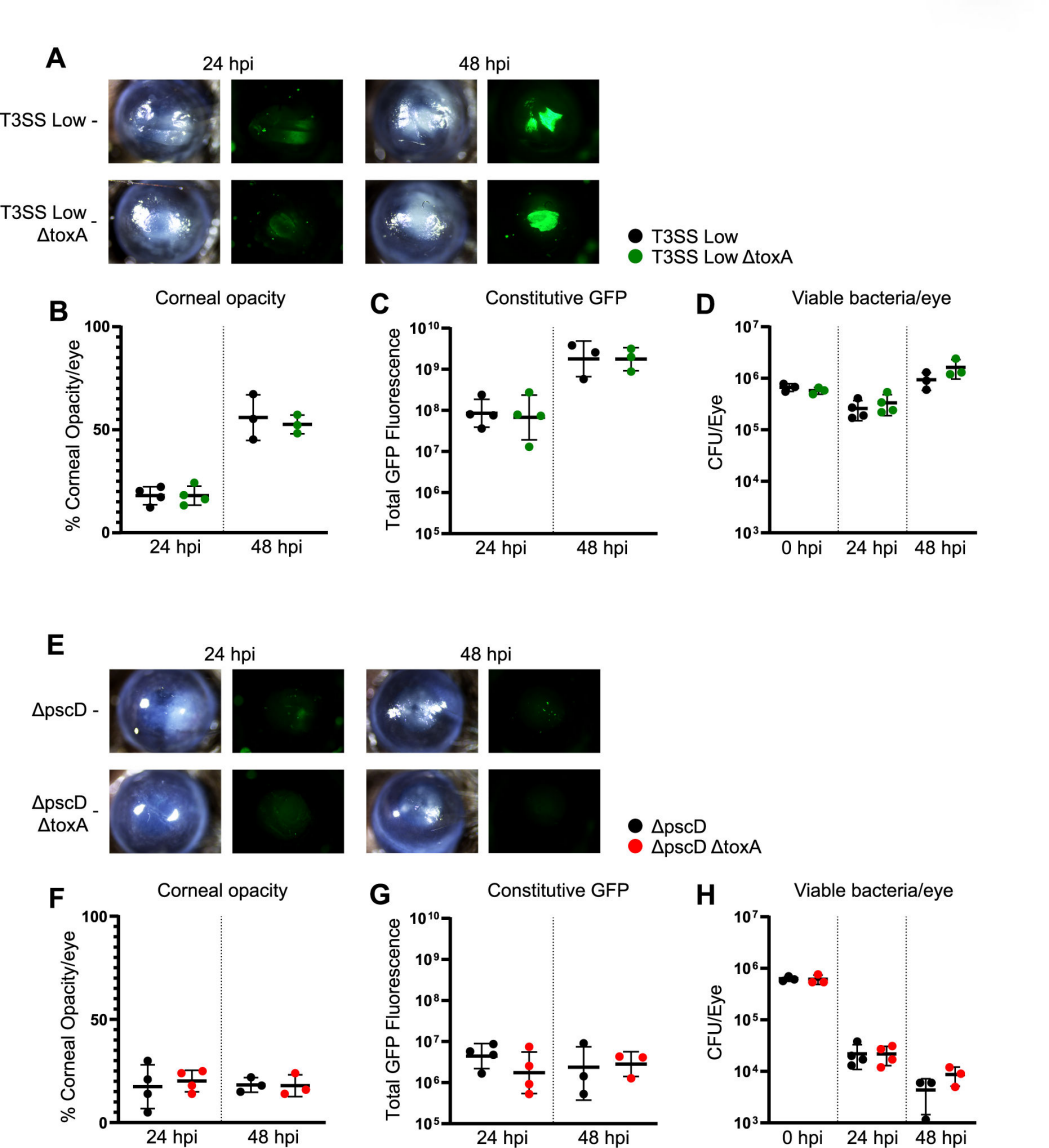
The effect of ToxA in T3SS-low and T3SS-deficient backgrounds. (**A–D**) Mice were infected with the T3SS-low PAO1 strain RP289 or Δ*toxA* strains on this background. (**E–H**) Mice were infected with PAO1F Δ*pscD* or Δ*pscD* Δ*toxA* strains. (**A–E**) Representative brightfield and constitutive GFP fluorescence images of infected corneas. (**B and F**) Corneal opacity quantified and expressed as a percentage. (**C and G**) Constitutive bacterial GFP fluorescence quantified from infected corneas. (**D and H**) Viable bacteria were measured by colony-forming units (CFU) from homogenized eyes. Each data point represents a single infected cornea. Error bars represent mean ± SD. Statistical analysis was performed using one-way ANOVA. *P* > 0.05 for all comparisons.

We found that this strain caused corneal opacification at 24h and 48h post-infection; however, there were no significant differences in corneal opacity, total GFP fluorescence, or bacterial growth between RP289 and RP289 ∆*toxA* strains (Fig. 4A through D). The Δ*pscD* T3SS deletion mutant caused mild infection, as we previously reported (21); however, *toxA* deletion in this background did not significantly alter disease severity or CFU recovery (Fig. 4E through H). Representative brightfield and GFP images from one experiment with the T3SS-low and Δ*pscD* infection experiments are shown in Fig. S5. Therefore, even with low T3SS activity and in Δ*pscD*, ToxA had no detectable effect on corneal opacity or bacterial growth.

## DISCUSSION

In this study, we tested whether Exotoxin A contributes to the pathogenesis of *P. aeruginosa* keratitis. We demonstrated using ToxA/GFP reporter strain and immunoblotting that ToxA is produced during corneal infection; however, while deletion of *toxA* reduced cytotoxic activity against human corneal epithelial cells *in vitro*, loss of ToxA did not inhibit corneal opacity, cellular infiltration, or bacterial growth in infected corneas. These findings indicate that, in the PAO1 keratitis model, ToxA is not a major determinant of corneal disease severity.

ToxA is a 66-kDa A-B toxin secreted as part of the T2SS and is mechanistically analogous to diphtheria toxin. The ToxA B (binding) domain attaches to the host cell surface receptor LRP1 (CD91), initiating receptor-mediated endocytosis (22). Following translocation into the cytosol, the catalytic A domain ADP-ribosylates eukaryotic elongation factor-2 (eEF-2), halting protein synthesis and inducing rapid cell death (23).

Iglewski et al. showed that ToxA is an important virulence factor in *P. aeruginosa* keratitis caused by PAO1 or the ExoU-expressing strains PA103 using a chronic model of corneal infection (24). Subsequently, Pillar and Hobden reported that Δ*toxA* mutants in PAO1 and ExoU-expressing 19660 were rapidly cleared from the cornea and resulted in reduced disease severity compared with parent strains (17). Using isogenic mutants generated in both PAO1 and 19660 backgrounds, these investigators demonstrated that ToxA was not required for bacterial adherence to wounded corneal epithelium or for initiation of infection, but was critical for bacterial persistence, sustained inflammation, and progression to severe disease. Over the past two decades, the Pillar and Hobden study has been cited extensively, and the prevailing view is that ToxA plays a major role in *P. aeruginosa* keratitis.

We used a similar well-established murine model that identified an important role of the T3SS in *P. aeruginosa* keratitis, including (9, 19, 21, 25). These include a role for ExoS and ExoT ADP ribosyl transferase (ADPRT) in inhibiting ROS production, ADP ribosylation of Ras that inhibits assembly of the NADPH oxidase complex (25). We also demonstrated that ExoS ADPRT activity confers NLRP3 inflammasome usage in infected neutrophils compared with NLRC4 usage in macrophages (9). In the current study, we determined if ToxA contributes to disease in a PAO1F background and in other strains that have either lower or no T3SS activity.

Using this approach, we found that the ToxA mutation had no effect on corneal disease across each PAO1F background tested. Although we confirmed by GFP expression and by western blot that ToxA protein is produced in infected corneas, we observed no differences in bacterial growth, disease severity, or inflammatory cell recruitment between PAO1F and Δ*toxA* strains. Despite matching the inoculum, infection method, mouse strain and age, and similar bacterial growth conditions reported by Pillar and Hobden (17), Δ*toxA* mutants did not show reduced virulence, which is distinct from this earlier report. We conclude that ToxA is not required for disease progression under the conditions tested in this model.

A possible difference between the present study and the study by Pillar and Hobden is the strain background examined. Iglewski et al. reported that strain PA103 produced 10-fold more ToxA than PAO1. In addition to PAO1, Pillar and Hobden examined strain 19660, an ExoU and ExoT-producing strain (17). ExoU is a highly cytotoxic phospholipase, and ExoU-producing strains typically cause more rapid and severe corneal disease than ExoS-producing strains such as PAO1 (4). Pillar and Hobden also showed lower myeloperoxidase production in Δ*toxA*-infected corneas, indicating fewer neutrophils; however, we used flow cytometry to demonstrate that there was no significant difference in recruitment of neutrophils and inflammatory monocytes. The underlying reasons for these differences between their findings and ours have therefore yet to be determined.

eEF-2-targeting toxins, including ToxA, activate the NLRP1 inflammasome and disrupt epithelial barrier integrity in human cells (26). Mechanistically, eEF-2 inhibition induces translational arrest and ribosome stalling, activating the ZAKα-dependent ribotoxic stress response and downstream p38 signaling, which in turn promotes NLRP1 activation through phosphorylation of a primate-specific N-terminal regulatory region (27–30). ZAKα-activating ribotoxins such as anisomycin can activate human NLRP1, but not murine NLRP1B, as anisomycin fails to induce NLRP1-dependent IL-1β release in murine keratinocytes and macrophages (28, 30, 31). Thus, ribotoxic stress, and therefore ToxA, may not induce the same inflammatory response in mice as in humans because NLRP1 is functionally distinct in primates and mice.

If ToxA activates a primate-specific NLRP1 pathway, then the relevant disease mechanism may need to be tested in the human cells that drive corneal inflammation. In the cornea, disease severity caused by *P. aeruginosa* is driven largely by recruited neutrophils in infected patients (4, 7). Inflammasome activation by ribotoxic stress in human neutrophils may represent an important mechanism by which ToxA contributes to disease. Future studies using human neutrophil and corneal epithelial cell models will be needed to determine whether ToxA-dependent ribotoxic stress drives a primate-specific inflammatory response that is not captured by murine models but may contribute to human infection.

Taken together, these findings indicate that ToxA is not a required determinant of corneal pathology in mice and highlight the importance of evaluating ToxA function in human cell-based models of corneal inflammation.

## SUPPLEMENTAL MATERIAL

Supplemental material iai.00165-26-s0001.pdf Table S1; Fig. S1 to S5.

ASM does not own the copyrights to Supplemental Material that may be linked to, or accessed through, an article. The authors have granted ASM a non-exclusive, world-wide license to publish the Supplemental Material files. Please contact the corresponding author directly for reuse.
