# Supplementary material for: Pseudomonas aeruginosa exotoxin A is not essential for corneal disease severity or bacterial survival
Source: Infect Immun. 2026 Jul 22;94(8):e00165-26. doi: 10.1128/iai.00165-26 (PMC13459711; doi:10.1128/iai.00165-26)
Supplement: Supplementary file 1 [file iai.00165-26-s0001.pdf]

# Supplemental Table 1

| | | |
|---|---|---|
| toxA-5-1G | CACGATCATGCGCACCCGTGGAAATTAATTAAGGTACCGGTGCACCGCACACGGCGGAG | toxA deleting codons 4-636, outside primer for Gibson cloning into pEXG2 KpnI/III |
| toxA-5-2 | CGGTCGCGGCAGTTACTTCAGCAGGTGCATCGCGATGGCTCCTTTGATGG | |
| toxA-3-1 | CATCAAAGGAGCCATCGCGATGCACCTGCTGAAGTAACTGCCGCGACCG | |
| toxA-3-2G | TTATACGAGCCGGAAGCATAAATGTAAAGCAAGCTTGAGGACAGCTCGGCGTAGACC | |
| ptoxA-5G | GCTAATTCGATCATGCATGAGCTCACTAGTggatccTCGACAATGCTCTCTCCGCCTG | clone ptoxA-toxA into Tn7T-Gmvector BamHI/HindIII cut |
| toxA-3G | CCTGCAAGGCCTTCGCGAGGTACCGGGCCCaagcttCGCGGCAGTTACTTCAGGTCCTC | |
| xcpQ3-1 | TTCAGCATGCTTGCGGCTCGAGTTGAATAACGGCGCCATACCGGCAAGGCGGCCTCA | internal xcpQ primer |
| xcpQ3-2 | AAAAAaagcttTGGCCAAGTACTGCACGCCTTATCT | HindIII site |
| xcpZ3-2 | AACTCGAGCCGCAAGCATGCTGAAGAGTGAGCGGCCCGTGGCTGGGGCCGGCCTG | internal xpcZ primer |
| xcpZ3-1 | AAAAAtctagaCAGGGGACGACGGAAACGAAAAAA | XbaI site |

**Table S1.** Primers used in this study.
Primers for toxA deleting codons 4–636 outside primer for Gibson cloning into pEXG2 KpnI/III and cloning ptoxA-toxA into the Tn7-Gm vector using BamHI/HindIII cut sites. Primers for deleting xcpQRSTUVWZYZ were used to amplify flanking regions and cloned conventionally into pEXG2 cut with XbaI and HindIII. Sequences are listed 5′→3′.

# Supplemental Figure 1

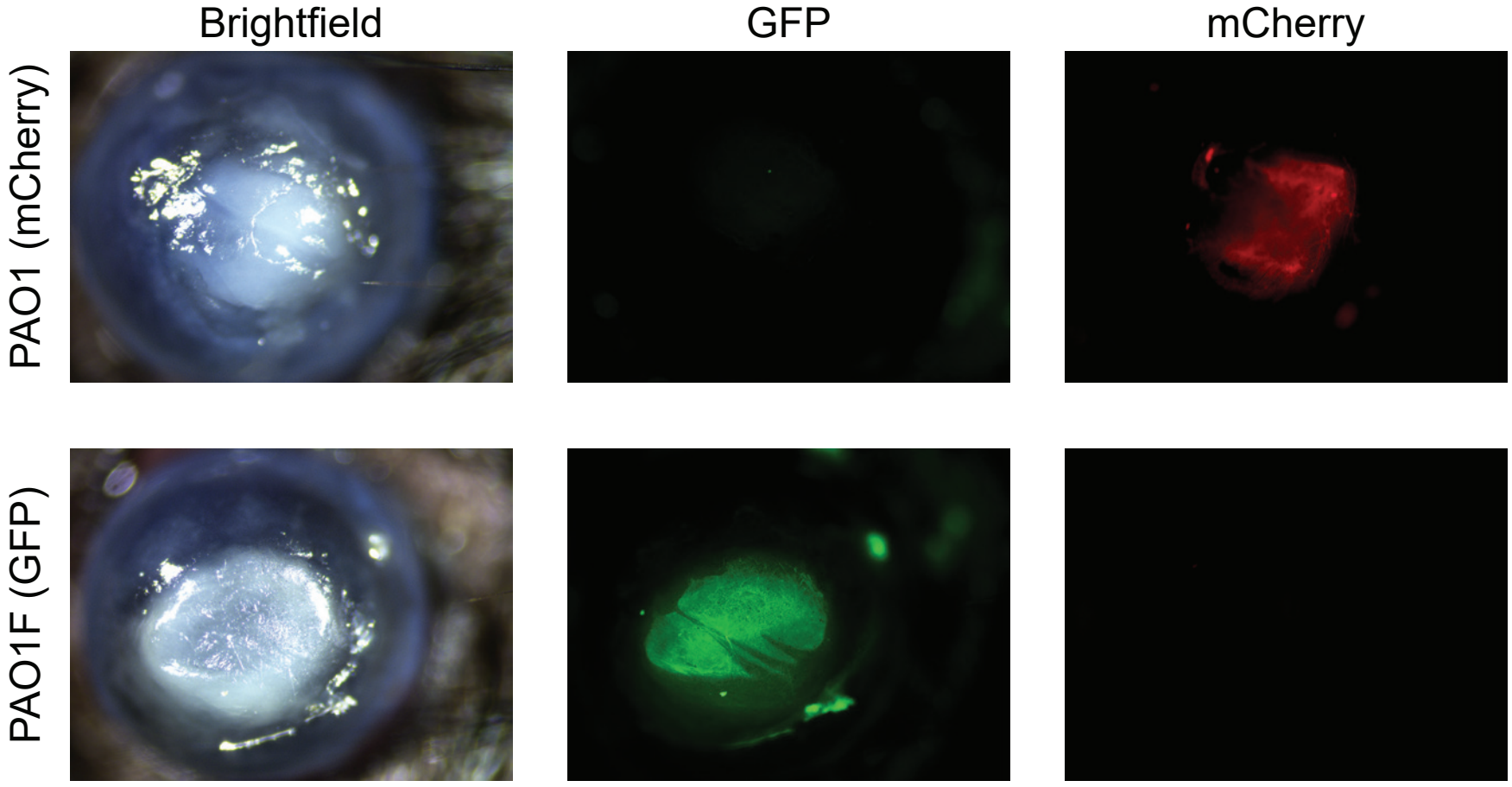


**Supplemental Figure 1.** No fluorescence bleed through between GFP and mCherry channels during corneal imaging. Representative images of infected corneas at 24 hours post-infection with *P. aeruginosa* strains expressing either constitutive mCherry alone (top row) or the GFP alone (bottom row). Corneas were imaged using brightfield (left), GFP channel (center), and mCherry channel (right). The mCherry only strain shows signal exclusively in the mCherry channel with no fluorescence in the GFP channel, indicating no mCherry to GFP bleed through. Conversely, the GFP reporter strain shows signal only in the GFP channel with no fluorescence in the mCherry channel, indicating no GFP to mCherry bleed through.

**Supplemental Figure 2**

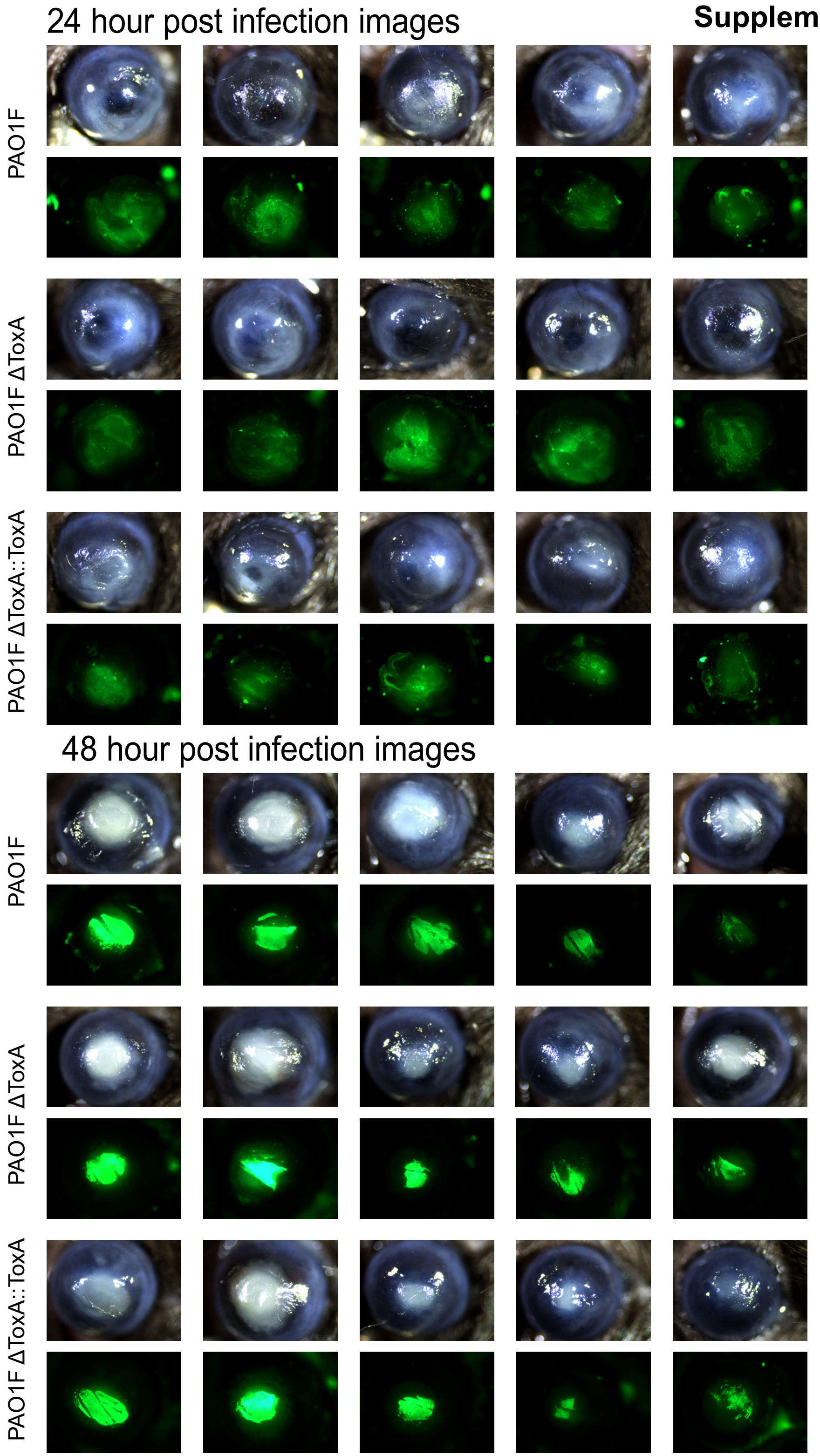


**Supplemental Figure 2.** Supporting corneal image set for Fig. 2
Brightfield and GFP fluorescence images of all corneas used for quantitative analysis in Fig. 2 following infection with PAO1F, ΔtoxA, or the complemented strain (ΔtoxA::toxA) at 24 and 48 hours post-infection. Each image corresponds to an individual eye included in opacity and GFP fluorescence quantification. These same eyes were subsequently collected for the CFU analysis seen in Fig. 2. This data supports that toxA deletion does not alter corneal pathology or bacterial growth in corneal disease.

# Supplemental Figure 3

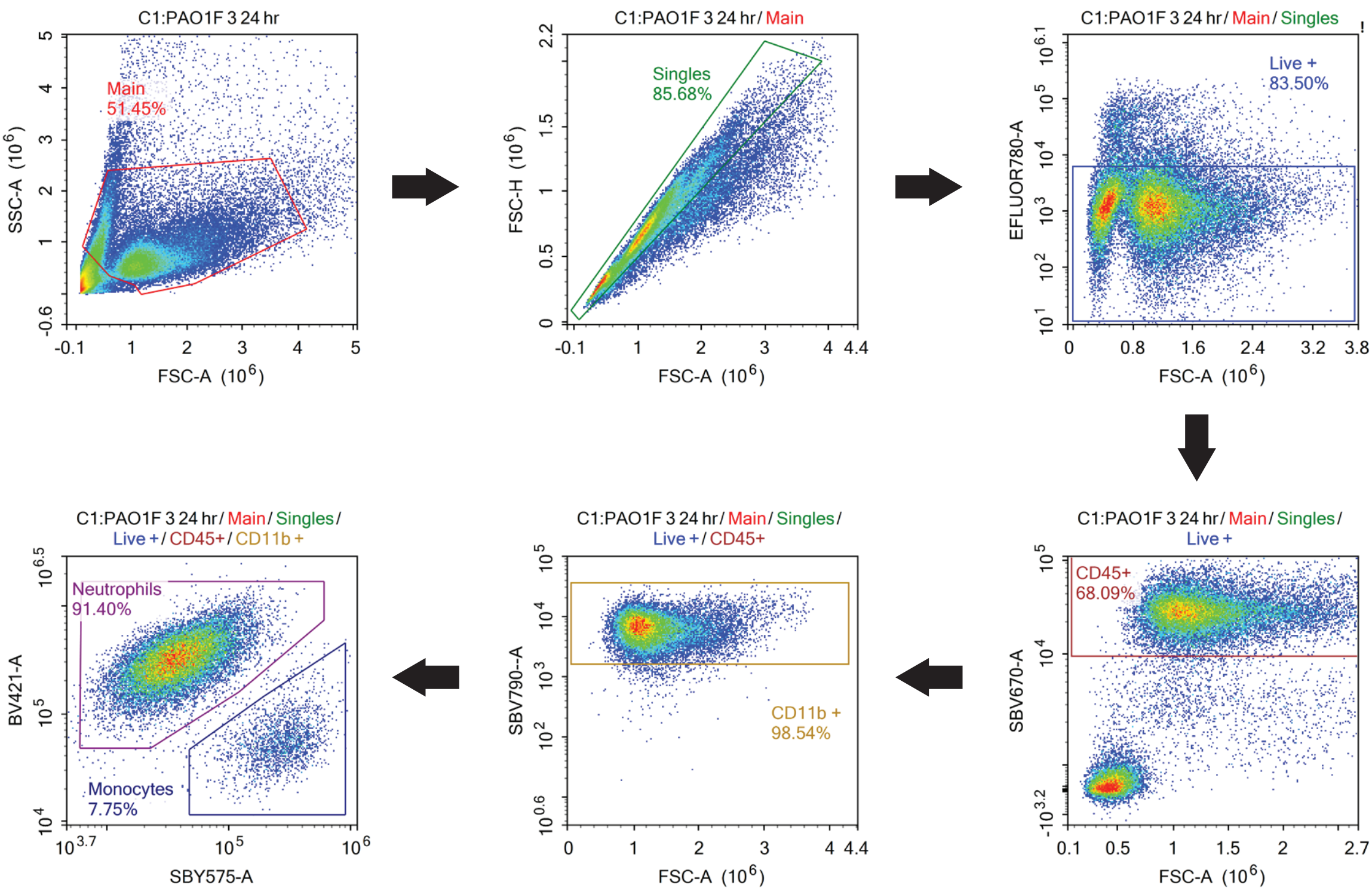


**Supplemental Figure 3.** Flow cytometry gating strategy supporting Fig. 3
Representative flow cytometry gating strategy used to identify leukocyte populations recovered from infected corneas. Cells were first gated on the main population by forward- and side-scatter, followed by singlets and live cells. Leukocytes were identified as $CD45^+$ cells, and myeloid cells were defined as $CD45^+CD11b^+$. Neutrophils were identified as $Ly6G^+$ cells and monocytes as $Ly6C^+$ cells within the $CD45^+CD11b^+$ population. This gating strategy was applied uniformly across all samples for the analyses shown in Fig. 3.

# Supplemental Figure 4

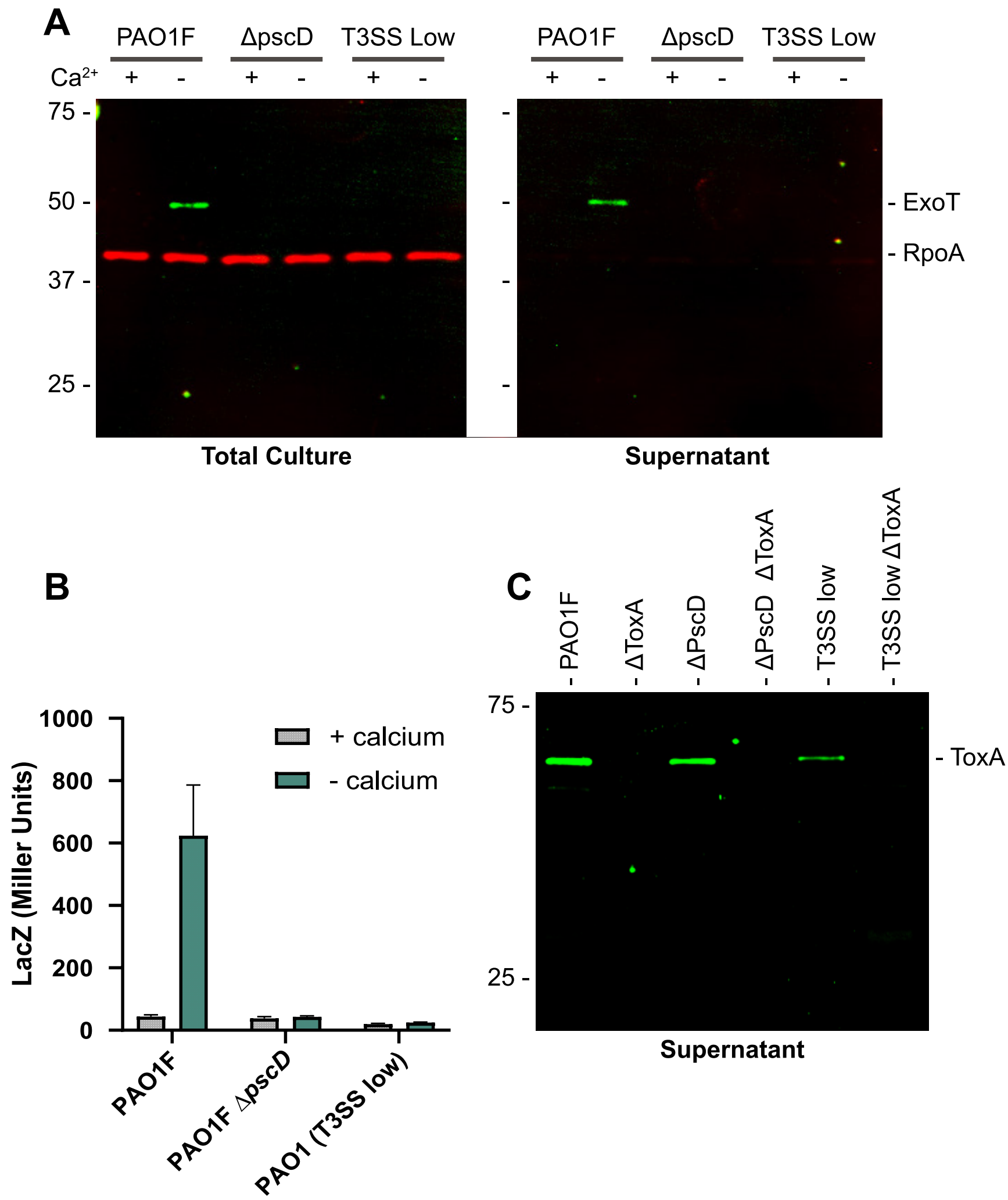


**Supplemental Figure 4.** Comparative characterization of PAO1F, ΔpscD, and T3SS-low strains.
**(A)** PAO1F, ΔpscD, and T3SS-low strains were grown in TSB-DF with or without $Ca^{2+}$. Total culture and supernatant fractions were analyzed by immunoblotting for ExoT, with RpoA measured as a total culture control. ExoT was detected in PAO1F under low-$Ca^{2+}$ conditions, but was not detected in PAO1F ΔpscD or T3SS-low PAO1. **(B)** β-galactosidase activity was measured in reporter strains harboring GFP followed by translationally coupled lacZ, where lacZ expression is driven by the exoS promoter and therefore reports exoS promoter activity. PAO1F showed increased β-galactosidase activity under low-$Ca^{2+}$ conditions, whereas PAO1F ΔpscD and T3SS-low PAO1 showed lower β-galactosidase activity. **(C)** Supernatants from PAO1F, ΔtoxA, ΔpscD, ΔpscD ΔtoxA, T3SS-low, and T3SS-low ΔtoxA were additionally probed for ToxA. ToxA was detected in the parent strains but not in the corresponding ΔtoxA mutants. These results provide comparative experimental characterization of the strains used in Fig. 4.

**Supplemental Figure 5**

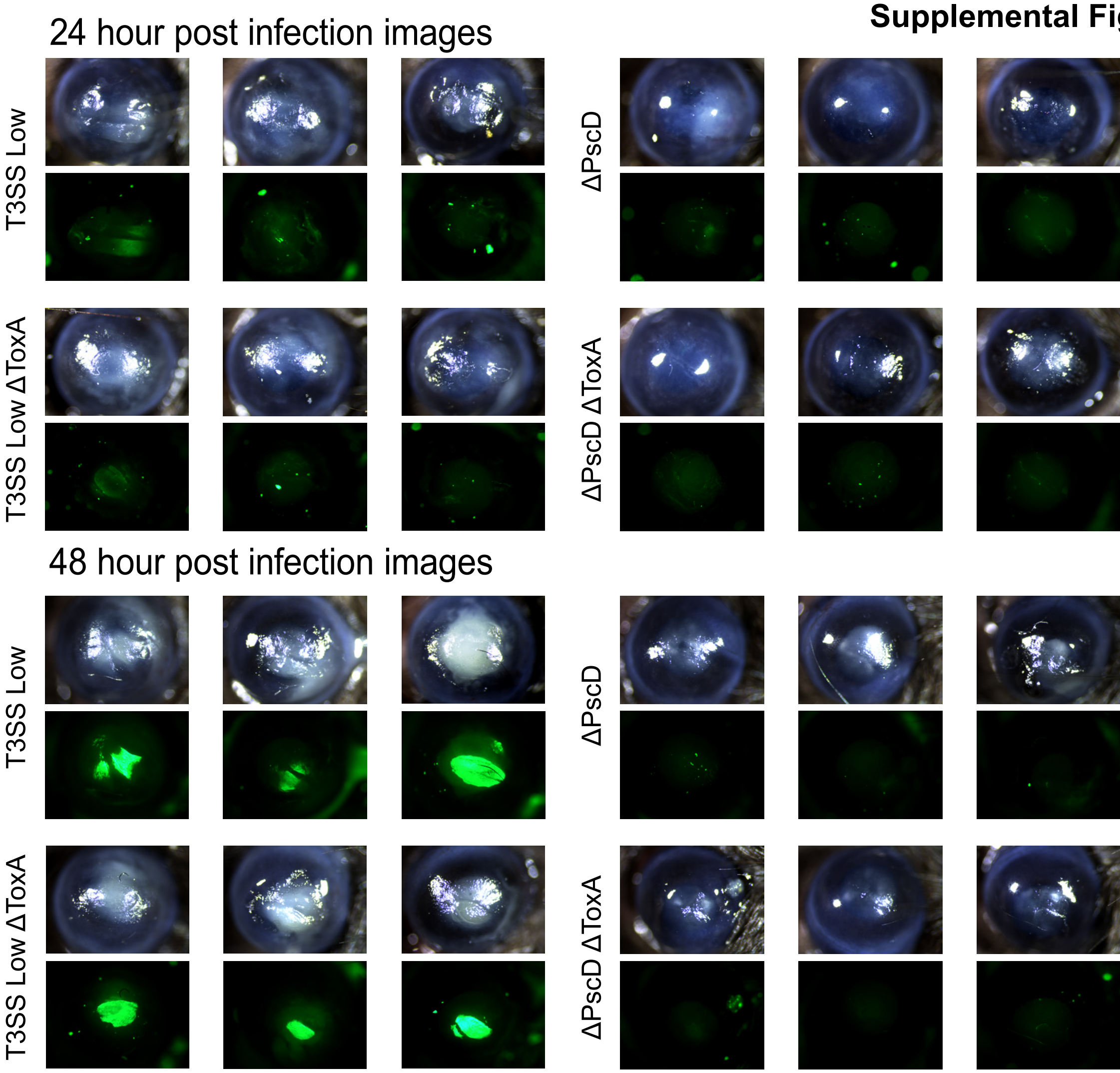


**Supplemental Figure 5.** Supporting corneal image set for Fig. 4
Brightfield and GFP fluorescence images of all corneas used for quantitative analysis in Fig. 4 following infection with T3SS-low strains or ΔpscD (T3SS-deficient) strains in the presence or absence of toxA at 24 and 48 hours post-infection. Multiple individual animals are shown to demonstrate consistency of disease severity and bacterial fluorescence across conditions. These images support the quantitative data presented in Fig. 4 and confirm that toxA deletion does not alter corneal pathology or bacterial growth in strains with reduced or absent type III secretion system activity.
